# Preoperative Risk Stratification of Acute Kidney Injury After Transcatheter Aortic Valve Implantation Using a Multimarker Model

**DOI:** 10.7759/cureus.112756

**Published:** 2026-07-15

**Authors:** Yumi Obata, Sachi Shimmi, Yusuke Seino, Atsuko Kamijo-Ikemori, Soichiro Inoue

**Affiliations:** 1 Department of Anesthesiology, St. Marianna University School of Medicine, Kawasaki, JPN; 2 Department of Anatomy, St. Marianna University School of Medicine, Kawasaki, JPN

**Keywords:** acute kidney injury, predictive model, risk prediction, transcatheter aortic valve implantation, urinary biomarker

## Abstract

Background

Acute kidney injury (AKI) following transcatheter aortic valve implantation (TAVI) is a major predictor of poor postoperative outcomes. Post-TAVI AKI is considered a multifactorial syndrome involving renal functional reserve, tubular injury, and systemic inflammation; however, reliable preoperative stratification methods remain limited. This study aimed to identify clinically relevant preoperative predictors reflecting these complementary pathophysiological domains and to evaluate their ability to stratify AKI risk before TAVI.

Methods

We retrospectively analyzed data from 186 patients undergoing TAVI under general anesthesia (AKI: 24 cases, 12.9%). Candidate predictors were selected based on previous studies and univariable analysis. Least absolute shrinkage and selection operator (LASSO) regression was used for variable selection. Model performance was assessed using area under the receiver operating characteristic curve (AUC), net reclassification improvement (NRI), integrated discrimination improvement (IDI), and decision curve analysis (DCA). Internal validation was performed using 200 bootstrap resamples.

Results

The final model incorporating preoperative estimated glomerular filtration rate (eGFR), urinary liver-type fatty acid-binding protein (L-FABP), clusterin, and C-reactive protein (CRP) demonstrated good discrimination for post-TAVI AKI (AUC=0.85). The model also showed improved risk reclassification (C-index 0.84, NRI 0.132, IDI 0.143) and favorable net clinical benefit in decision curve analysis, particularly at low-risk thresholds (0.10-0.20). These variables reflect distinct biological domains, including renal functional reserve, tubular injury, and systemic inflammation.

Conclusion

A preoperative risk stratification model integrating urinary biomarkers and inflammatory and renal functional markers may facilitate the identification of patients with latent renal vulnerability before TAVI. Post-TAVI AKI appears to arise from multiple interrelated pathophysiological processes rather than isolated renal dysfunction.

## Introduction

Acute kidney injury (AKI) following transcatheter aortic valve implantation (TAVI) is associated with poor prognosis; however, a highly accurate preoperative predictive method has not yet been established [1-3]. Preoperative clinical factors, such as chronic kidney disease, diabetes, hypoalbuminemia, and reduced cardiac function, have traditionally been used to evaluate post-TAVI AKI risk [3-5]. However, these clinical factors alone cannot sufficiently predict postoperative AKI, and speculating on the development of AKI based solely on estimated glomerular filtration rate (eGFR) is challenging. Furthermore, as the diagnosis of AKI using the Kidney Disease: Improving Global Outcome (KDIGO) criteria is confirmed only after renal function decline, therapeutic intervention is frequently delayed [6]. In elderly patients undergoing TAVI, this delay poses risks for chronic kidney disease progression and dialysis [7]. Therefore, appropriate stratification of AKI risk before renal function decline is essential to enable early intervention.

Biomarkers are increasingly used to diagnose AKI [8]. We previously reported that novel urinary biomarkers, including liver-type fatty acid-binding protein (L-FABP) and clusterin, signify renal ischemia and tubular injury at an early stage and may provide information beyond the conventional renal function indices [9]. The majority of patients undergoing TAVI are elderly, and in this population, AKI pathogenesis is multifactorial, involving overlapping mechanisms, such as ischemia, inflammation, and oxidative stress [10,11]. This explains why single clinical factors capture only one aspect of AKI pathology, thereby limiting predictive performance. Although diagnostic accuracy can be improved using multiple urinary biomarkers, incorporating biomarkers with other clinical information is likely necessary to achieve higher predictive accuracy [12]. However, a predictive model incorporating preoperative urinary biomarkers with clinical factors has not yet been established [9,12].

We hypothesized that incorporating renal ischemia and tubular injury signals captured by urinary biomarkers with information provided by clinical factors, such as renal reserve, systemic inflammation, and nutritional status (including hypoalbuminemia), would enhance the prediction of post-TAVI AKI, which develops through a multifactorial mechanism. Because individual biomarkers and clinical variables reflect different aspects of AKI pathophysiology, integrating these complementary factors may improve preoperative risk stratification. Therefore, we applied a Least Absolute Shrinkage and Selection Operator (LASSO)-regularized logistic regression approach to identify the most relevant predictors among the urinary biomarkers and clinical variables and evaluated their combined predictive performance. The aim of this study was to evaluate whether integrating urinary biomarkers with conventional clinical variables using a LASSO-regularized logistic regression approach could improve the preoperative risk stratification of post-TAVI AKI.

## Materials and methods

Study design

This retrospective observational study was conducted as a post-hoc analysis of a previously established cohort of patients who underwent TAVI at a single-center university hospital between December 2016 and June 2020. Data from 186 patients were analyzed. From an initial cohort of 435 cases, patients classified as emergency cases, receiving dialysis, or requiring conversion to open-heart surgery due to intraoperative complications, such as annulus rupture or aortic dissection, were excluded. All included patients received anesthesia care provided by the authors. Of the 186 patients included in the present study, 173 were part of a previously reported cohort used to evaluate individual urinary biomarkers [9]. Owing to funding limitations, 13 eligible patients had not been analyzed in the previous study and were subsequently included, resulting in the complete cohort of 186 patients in the present investigation. Because this was a retrospective study, postoperative serum creatinine measurements were obtained from routine clinical practice rather than according to a predefined study protocol. Consequently, serum creatinine was not measured on postoperative day 2 in some patients.

The study protocol was approved by the Ethics Committee of our institution on September 18, 2025 (approval no. 7002) and registered in the UMIN Clinical Trials Registry (ID: UMIN000058857). The study was conducted in accordance with the principles of the Declaration of Helsinki (last revised 2013) as well as all applicable national laws and institutional guidelines. To protect confidentiality and ensure data privacy, patient data were analyzed using de-identified codes.

TAVI procedure and anesthesia management

TAVI procedures were performed under general anesthesia with endotracheal intubation using the transfemoral approach. Balloon- or self-expandable valves were selected based on clinical indication. Standard intraoperative monitoring, including transesophageal echocardiography and continuous noradrenaline infusion, was performed at the anesthesiologist’s discretion.

Data collection and renal function evaluation

Baseline characteristics (sex, age, body mass index (BMI), comorbidities, medication), results of preoperative laboratory tests (hemoglobin, serum albumin, blood glucose, serum electrolytes, serum creatinine (sCr), white blood cell count, C-reactive protein (CRP), N-terminal pro B-type natriuretic peptide), echocardiographic findings (left ventricular ejection fraction (LVEF), aortic valvular mean gradient), and intraoperative data were collected from the hospital electronic medical and anesthetic records. Blood and urine samples were collected at hospital admission, which occurred within three days before the TAVI procedure in most patients.

Postoperative serum samples were obtained at the following time points: immediately after the procedure and on postoperative days (PODs) 1 to 7. The sCr levels were determined via an enzymatic assay. Upon hospital admission for surgery, the eGFR was calculated using the modified Chronic Kidney Disease Epidemiology Collaboration equation, adjusted with the Japanese coefficient [13]:

$\mathrm{eGFR}=194 \times (\mathrm{Creatinine})^{-1.094}\times (\mathrm{Age})^{-0.287}\times (0.739\ \text{if female})$

Serum albumin and CRP levels were measured in the central laboratory via standard automated assays. Urinary L-FABP level was measured via a latex-enhanced immunoturbidimetric assay with antihuman L-FABP mouse monoclonal antibodies and the urinary clusterin level was measured using the Human Clusterin Quantikine enzyme-linked immunosorbent assay (ELISA) kit. All urinary biomarker analyses were conducted by a laboratory technician (not involved in the clinical assessments). Other laboratory measurements were performed in the central laboratory, unless otherwise specified.

Diagnosis of post-TAVI AKI

AKI was defined according to the KDIGO criteria [6]. The diagnostic window was extended from 72 h to POD 7. AKI staging was determined based on sCr elevation and urine output criteria.

Development of predictive models

Multivariable logistic regression models were developed for post-TAVI AKI prediction. To minimize overfitting considering a limited number of AKI events (n=24), variable selection was performed using logistic regression with the least absolute shrinkage and selection operator (LASSO) regularization applied to preoperative candidate predictors. Candidate variables for LASSO logistic regression were selected based on univariable associations in the present cohort, previous literature, and clinical plausibility. The candidate variables entered into the LASSO logistic regression included age, sex, diabetes mellitus, hypertension, ischemic heart disease, chronic obstructive pulmonary disease, hyperlipidemia, β-blocker use, calcium channel blocker use, angiotensin receptor blocker use, diuretic use, nonsteroidal anti-inflammatory drug use, ejection fraction, mean pressure gradient, body mass index, preoperative estimated glomerular filtration rate, serum albumin, calcium, chloride, potassium, sodium, white blood cell count, glucose, C-reactive protein, hemoglobin, hematocrit, N-terminal pro B-type natriuretic peptide (NT-proBNP), urinary N-acetyl-β-D-glucosaminidase, urinary liver-type fatty acid-binding protein-to-creatinine ratio, urinary clusterin-to-creatinine ratio, and urinary tissue inhibitor of metalloproteinases-2-to-creatinine ratio. Categorical variables were coded as binary variables, and continuous variables were analyzed as continuous predictors. Urinary biomarker variables were transformed using log(1+x) before model development to account for skewed distributions. Model complexity was restricted to less than three degrees of freedom. Multivariable logistic regression models were constructed using variables selected by the LASSO. Missing data were handled via multiple imputation, and the entire modeling procedure was performed independently within each imputed dataset (m=20). Optimal penalization parameter (λ) was determined via stratified 10-fold cross-validation to account for outcome imbalance. To prioritize model parsimony and generalizability, the one-standard-error rule (λ₁se) was mainly adopted, selecting the simplest model whose prediction error was within one standard error of the minimum.

Based on the LASSO selection results across the imputed datasets, renal function markers (preoperative eGFR (pre-eGFR)), selected urinary biomarkers reflecting tubular injury (urinary L-FABP and clusterin), and an inflammatory marker (preoperative CRP (pre-CRP)) were repeatedly identified as non-zero coefficient predictors. Multiple candidate predictive models were developed by incorporating these variables while considering LASSO-based selection frequency and clinical plausibility.

For comparison, two reference models were defined a priori. Model 1 included pre-eGFR alone. Model 2 represented a conventional clinical model incorporating age, BMI, and LVEF, selected based on previous literature rather than the LASSO results. Additional models (Models 3-19) were developed by sequentially adding and recombining urinary biomarkers and inflammatory markers frequently selected by LASSO, aiming to capture complementary glomerular and tubular injury mechanisms as well as systemic inflammation.

Statistical analysis

Continuous variables were evaluated for normality using the Kolmogorov-Smirnov test. Normally distributed variables were expressed as mean±standard deviations and compared using Student’s t-test, whereas non-normally distributed variables, including urinary L-FABP and clusterin, were log-transformed and compared using the Mann-Whitney U test. Categorical variables were analyzed using the chi-squared test. Candidate predictors of post-TAVI AKI were selected based on univariate associations in the present cohort (p<0.10) and previous literature. Missing data were handled via multivariate imputation using chained equations, assuming that the data were missing at random. Continuous variables were imputed via predictive mean matching; no binary variable required imputation.

To evaluate the balance of baseline characteristics between groups with different sample sizes, absolute standardized differences (ASD) were calculated for all continuous and categorical variables. Continuous variables were standardized using the pooled standard deviation, whereas categorical variables were standardized according to the pooled proportion. ASD<0.1 indicated good balance, whereas values ≥0.2 indicated potential imbalance. The ASD was reported as a supplementary measure alongside p-values, and all multivariable analyses were adjusted for potential confounders.

Model performance was evaluated for each prespecified candidate model (Models 1-19). Logistic regression models were separately fitted within each of the multiple imputed datasets (m=20), and apparent model performance was assessed in each dataset. Discrimination was mainly evaluated using the area under the receiver operating characteristic curve (AUC). As the AUC is numerically equivalent to Harrell’s concordance index (C-index) for binary outcomes, discrimination results are also reported as C-index. Overall predictive accuracy was assessed using the Brier score, mean absolute error, 90th percentile absolute error, maximum absolute error, and area under the precision-recall curve.

Calibration was evaluated using calibration plots as well as the calibration intercept and slope, estimated based on the logit of the predicted probabilities. All performance metrics were summarized as mean values with corresponding standard deviations across the 20 imputed datasets. To account for potential overfitting, internal validation was performed via bootstrap resampling with 200 repetitions. In each bootstrap sample, models were fully refitted and optimism-corrected estimates of discrimination (C-index) and calibration slope were derived. Apparent and optimism-corrected performance measures were reported separately. The predictive performance enhancement between models was further evaluated using net reclassification improvement (NRI) and integrated discrimination improvement (IDI). Category-based NRI was evaluated using prespecified risk thresholds (e.g., 0.05-0.30). Clinical utility was assessed via decision curve analysis (DCA). All statistical analyses were conducted using IBM SPSS Statistics version 21.0 (IBM Corp, Armonk, NY) and the R software (R Foundation for Statistical Computing, Vienna, Austria). A two-sided p-value <0.05 was considered statistically significant. This study was conducted and reported in accordance with the TRIPOD (Transparent Reporting of a multivariable prediction model for Individual Prognosis Or Diagnosis) guidelines to ensure transparency and reproducibility.

## Results

Characteristics of the study cohort

**Figure 1:**
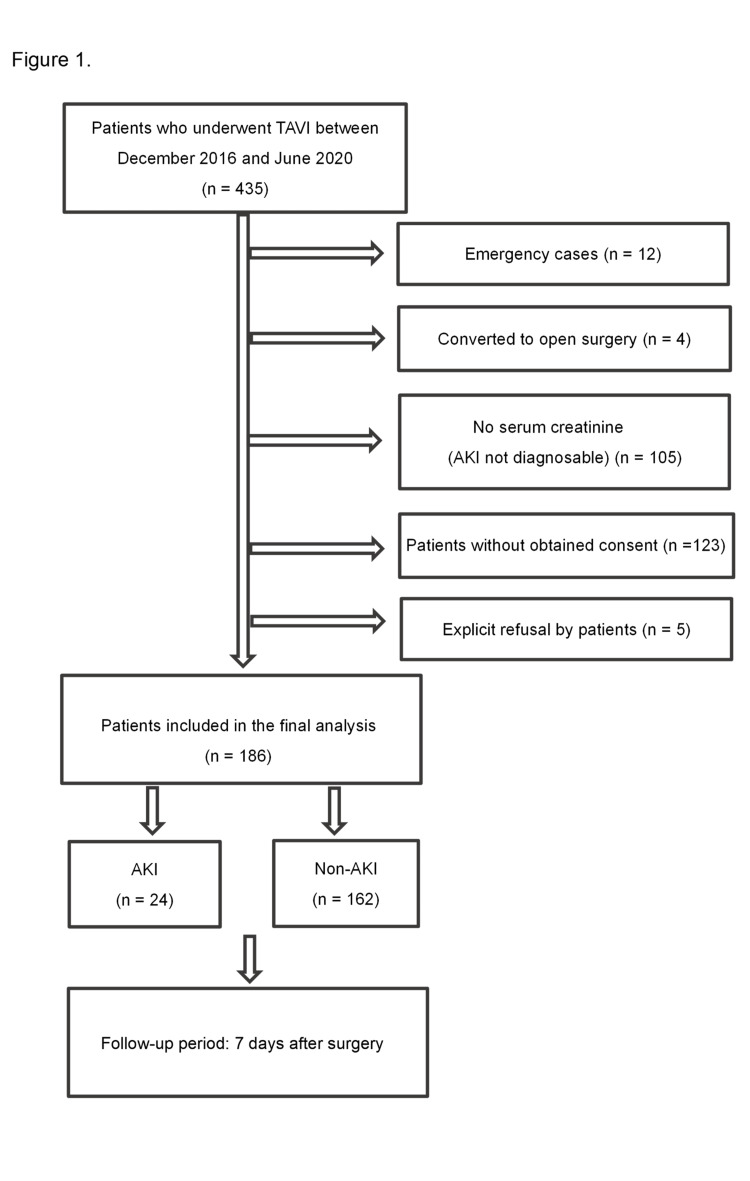
Patient flow diagram. A total of 435 patients underwent TAVI at our institution between December 2016 and June 2020. For the present study, we analyzed only the 186 patients in whom the author (Dr. Yumi Obata) was directly responsible for anesthetic management. Patients were excluded for the following reasons: emergency cases (n=12), conversion to open surgery (n=4), missing creatinine data (n=105), lack of consent (n=123), or explicit refusal (n=5). A preliminary analysis involving 173 patients was previously published; data from the remaining 13 patients were analyzed later to complete the full 186-case dataset. TAVI: Transcatheter aortic valve implantation.

Among the 186 patients included in the study, 24 (12.9%) developed post-TAVI AKI, most of whom had stage 1 AKI (Figure 1). The baseline characteristics of the patients are summarized in Table 1. Patients who developed AKI had significantly higher preoperative urinary L-FABP and clusterin levels than those without AKI. The diagnostic performance of preoperative urinary biomarkers is presented in Table 2.

**Table 1:** Patient characteristics Data are expressed as mean (SD), median (interquartile range) or number (%). *p<0.05. AKI, acute kidney injury; ASD, absolute standardized difference; BMI, body mass index; ACE, inhibitor angiotensin-converting enzyme inhibitor; ARB, angiotensin II receptor blocker; NSAIDs, nonsteroidal anti-inflammatory drugs; sCr, serum creatinine; eGFR, estimated glomerular filtration rate; WBC, white blood cell count; CRP, C-reactive protein, NT-pro BNP; N-terminal pro B-type natriuretic peptide; L-FABP, liver-type fatty acid–binding protein. eGFR was calculated according to the Japanese coefficient–modified Chronic Kidney Disease Epidemiology Collaboration equation [13]: $\mathrm{eGFR}=194 \times (\mathrm{Creatinine})^{-1.094}\times (\mathrm{Age})^{-0.287}\times (0.739\ \text{if female})$ Continuous variables with a normal distribution were compared using Student's t-test; non-normally distributed continuous variables were compared using the Mann-Whitney U test; categorical variables were compared using the chi-square test or Fisher's exact test, as appropriate.

	AKI group (n=24)	Non–AKI group (n=162)	Missing, n (%)	Statistic	P value	ASD
Sex (M/F), n	14/10	61/101		χ^2^=2.905	0.088	0.42
Age (years)	83.8 (5.1)	83.4 (5.3)		t=-0.319	0.750	0.07
BMI (kg/m^2^)	23.8 (3.7)	22.6 (3.7)	2 (1.1)	t=1.524	0.129	0.33
Left ventricular ejection fraction (%)	59.2 (11.1)	64.9 (10.9)		t=-2.413	0.017^*^	0.52
Mean gradient, mmHg	39 (26-46)	39 (25-55)		W=1826	0.633	0.18
Diabetes mellitus, n (%)	8 (33)	28 (17)		-	0.092	0.38
Hypertension, n (%)	24 (100)	131 (81)		-	0.016^*^	0.69
Ischemic heart disease, n (%)	9 (38)	54 (33)		χ^2^=0.0294	0.864	0.09
Chronic kidney disease, n (%)	24 (100)	117 (72)		χ^2^=7.345	0.007^*^	0.88
Stage 1	1 (4.2)	1 (0.9)		-	0.313	0.23
Stage 2	0 (0)	20 (17.1)		-	0.025^*^	0.53
Stage 3a	7 (29.2)	52 (44.4)		χ^2^=1.333	0.248	0.07
Stage 3b	6 (25.0)	33 (28.2)		χ^2^=0.00480	0.945	0.11
Stage 4	6 (25.0)	10 (8.5)		-	0.032^*^	0.54
Stage 5	4 (16.7)	1 (0.9)		-	0.003^*^	0.60
Medications, n (%)						
β-blocker	7 (29)	53 (33)		χ^2^=0.0128	0.910	0.77
Ca antagonist	14 (58)	72 (44)		χ^2^=1.112	0.292	0.28
ACE inhibitor/ARB	10 (42)	71 (44)		χ^2^=7.923	1.000	0.04
Statin	10 (42)	64 (40)		χ^2^=8.13×10⁻³²	1.000	0.04
Diuretic	7 (29)	42 (26)		χ^2^=0.00776	0.930	0.07
NSAIDs	0 (0)	1 (0.6)		-	1.000	0.11
Preoperative blood test results						
Hemoglobin Level (g/dL)	10.9 (1.7)	11.5 (1.7)	1 (0.5)	t=1.637	0.103	0.35
Serum albumin (g/dL)	3.36 (0.54)	3.81 (0.45)	1 (0.5)	t=-4.496	< 0.001^*^	0.92
Blood glucose (mg/dL)	116 (106-137)	110 (97-129)	5 (2.7)	W=2226	0.154	0.20
Serum sodium (mEq/L)	140 (138-141)	140 (138-141)	1 (0.5)	W=1948	0.949	0.03
Serum potassium (mEq/L)	4.15 (4.00-4.43)	4.10 (3.90-4.45)	1 (0.5)	W=1957	0.922	0.02
Serum chloride (mEq/L)	105 (103-108)	105 (102-107)	1 (0.5)	W=2154	0.364	0.28
Serum Cr (mg/dL)	1.30 (1.04–2.56)	0.84 (0.68-1.05)		W=3058	< 0.001^*^	0.90
eGFR (8mL/min/1.73m²)	36.0 (20.0)	55.2 (17.2)		t=-4.988	< 0.001^*^	1.03
WBC count (×10³/µL)	6.11 (3.02)	5.56 (1.64)	1 (0.5)	t=-0.882	0.386	0.23
CRP (mg/dL)	0.26 (0.07-1.02)	0.08 (0.03-0.26)		W=2669	0.003^*^	0.46
NT-proBNP (pg/mL)	1952 (942-4516)	895 (352-1748)	48 (25.8)	W=633	0.010^*^	0.69
Preoperative urinary parameters						
L-FABP (μg/gCr)	7.96 (2.56-55.3)	2.96 (1.86-5.22)	2 (1.1)	W=2530	0.005^*^	0.66
Clusterin (μg/gCr)	174.7 (49.5-1144.1)	17.0 (6.7-69.3)	3 (1.6)	W=2796	< 0.001^*^	0.73

**Table 2:** Diagnostic performance of urinary biomarkers and prediction models for post-TAVI AKI TAVI, transcatheter aortic valve implantation; AKI, acute kidney injury; AUC, area under the curve; CI, confidence interval; PPV, positive predictive value; NPV, negative predictive value; PLR, positive likelihood ratio; NLR, negative likelihood ratio; L-FABP, liver-type fatty acid–binding protein; Pre-op, pre-operation; eGFR, estimated glomerular filtration rate; CRP, C-reactive protein; $\mathrm{eGFR}=194 \times (\mathrm{Creatinine})^{-1.094}\times (\mathrm{Age})^{-0.287}\times (0.739\ \text{if female})$ [13]

Time point	AUC (95%CI)	Cut-off value	Sensitivity	Specificity	PPV	NPV	Diagnostic accuracy	PLR	NLR
Pre-ope eGFR	0.784 (0.679–0.890)	38.5	0.63	0.83	0.36	0.94	0.81	3.75	0.45
Pre-ope serum albumin	0.748 (0.645–0.852)	3.40	0.58	0.82	0.33	0.93	0.79	3.24	0.51
Pre-ope CRP	0.686 (0.581–0.792)	0.25	0.54	0.74	0.36	0.94	0.81	3.75	0.45
L-FABP (μg/gCr)									
Pre-operation	0.683 (0.543–0.823)	8.20	0.48	0.87	0.34	0.92	0.82	3.7	0.60
Clusterin (μg/gCr)									
Pre-operation	0.760 (0.638–0.881)	65.6	0.74	0.73	0.28	0.95	0.73	2.7	0.36

Prediction of post-TAVI acute kidney injury via LASSO logistic regression

Univariate logistic regression analyses revealed that LVEF, serum albumin, sCr, eGFR, CRP, log preoperative L-FABP (log pre-L-FABP), and log preoperative clusterin (log pre-clusterin) were significantly related to post-TAVI AKI. Serum albumin, eGFR, CRP, log pre-L-FABP, and log pre-clusterin were selected via LASSO regression (Table 3).

**Table 3:** Composition of the 19 Predictive Models Evaluated for Post-TAVI AKI TAVI, transcatheter aortic valve implantation; AKI, acute kidney injury; eGFR, estimated glomerular filtration rate; BMI, body mass index; EF, left ventricular ejection fraction; L-FABP, liver-type fatty acid–binding protein, Cr; serum creatinine, sAlb, serum albumin; CRP, C-reactive protein; Hb, hemoglobin.

Model	Variables
1	Pre-eGFR
2	Pre-eGFR + age + BMI + EF
3	Pre-eGFR + log(pre-L-FABP/Cr) + log(pre-clusterin/Cr) + pre-sAlb + EF + pre-CRP + pre-Hb
4	Pre-eGFR + EF + pre-sAlb + pre-CRP
5	Pre-eGFR + EF + pre-sAlb + pre-Hb
6	Pre-eGFR + EF + pre-CRP + pre-Hb
7	Pre-eGFR + pre-sAlb + pre-CRP + pre-Hb
8	Pre-eGFR + log(pre-L-FABP/Cr) + log(pre-clusterin/Cr) + EF
9	Pre-eGFR + log(pre-L-FABP/Cr) + log(pre-clusterin/Cr) + pre-Hb
10	Pre-eGFR + log(pre-L-FABP/Cr) + log(pre-clusterin/Cr) + pre-sAlb
11	Pre-eGFR + log(pre-L-FABP/Cr) + log(pre-clusterin/Cr) + pre-CRP
12	Pre-eGFR + log(pre-clusterin/Cr) + EF
13	Pre-eGFR + log(pre-clusterin/Cr) + pre-Hb
14	Pre-eGFR + log(pre-clusterin/Cr) + pre-sAlb
15	Pre-eGFR + log(pre-clusterin/Cr) + pre-CRP
16	Pre-eGFR + log(pre-L-FABP/Cr) + EF
17	Pre-eGFR + log(pre-L-FABP/Cr) + pre-Hb
18	Pre-eGFR + log(pre-L-FABP/Cr) + pre-sAlb
19	Pre-eGFR + log(pre-L-FABP/Cr) + pre-CRP

**Figure 2:**
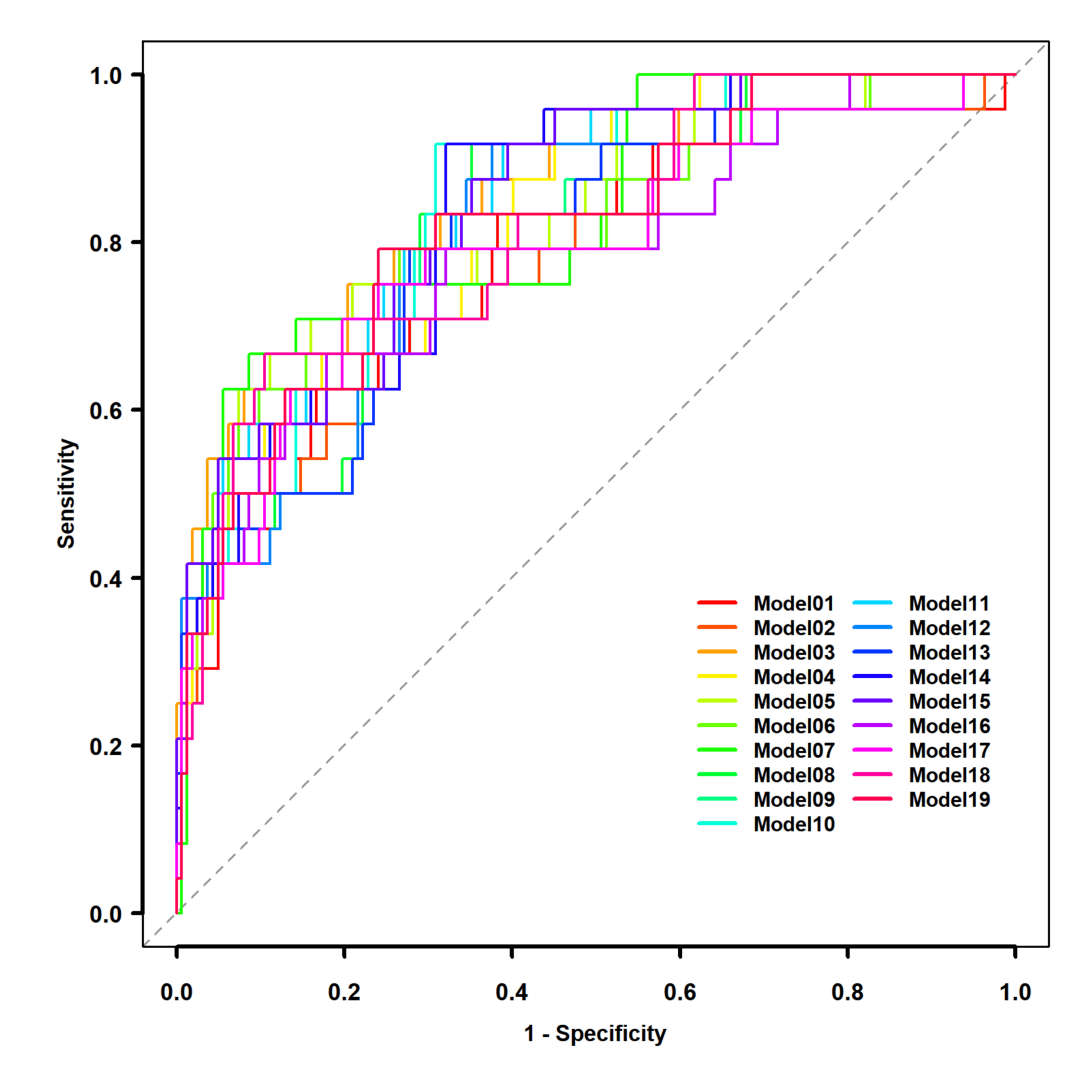
Receiver operating characteristic (ROC) curves for all 19 acute kidney injury predictive models included in the internal validation. The discriminative performance of 19 predictive models was evaluated to assess their ability to predict AKI following transcatheter aortic valve implantation (TAVI). Model 1 includes the preoperative estimated glomerular filtration rate (eGFR) alone. Model 2 is a conventional clinical model consisting of eGFR, age, body mass index, and left ventricular ejection fraction. Model 3 is a full model incorporating all variables, including eGFR, urinary biomarkers (liver-type fatty acid–binding protein [L-FABP] and clusterin), serum albumin, EF, C-reactive protein, and hemoglobin. Models 4-7 are simplified models incorporating eGFR with conventional clinical variables. Models 8-19 evaluate combinations of eGFR with urinary L-FABP and/or clusterin and a single clinical variable. Internal validation was performed using the bootstrap method. Model compositions are summarized in Table 4. .

**Figure 3:**
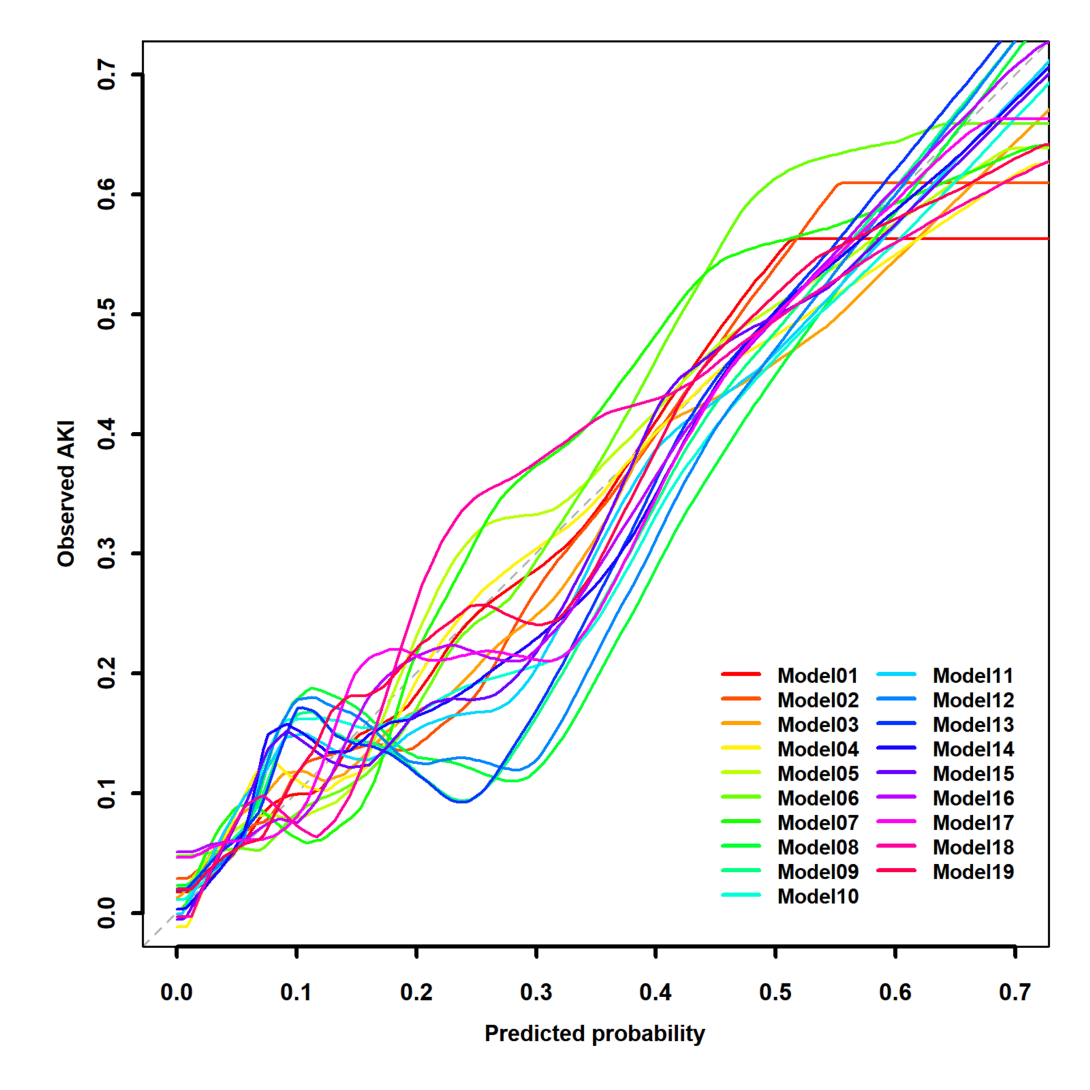
Calibration plots for all 19 acute kidney injury predictive models included in the internal validation. Calibration plots were used to evaluate the agreement between the predicted and observed probabilities of acute kidney injury (AKI) following transcatheter aortic valve implantation (TAVI) for 19 predictive models. Model 1 includes the preoperative estimated glomerular filtration rate (eGFR) alone. Model 2 is a conventional clinical model consisting of eGFR, age, body mass index, and left ventricular ejection fraction. Model 3 is a full model incorporating all variables, including eGFR, urinary biomarkers (liver-type fatty acid–binding protein (L-FABP) and clusterin), serum albumin, ejection fraction (EF), C-reactive protein, and hemoglobin. Models 4-7 are simplified models combining eGFR with conventional clinical variables. Models 8-19 evaluate combinations of eGFR with urinary L-FABP and/or clusterin and a single clinical variable. Internal validation was performed using the bootstrap method. Model compositions are summarized in Table 4.

**Figure 4:**
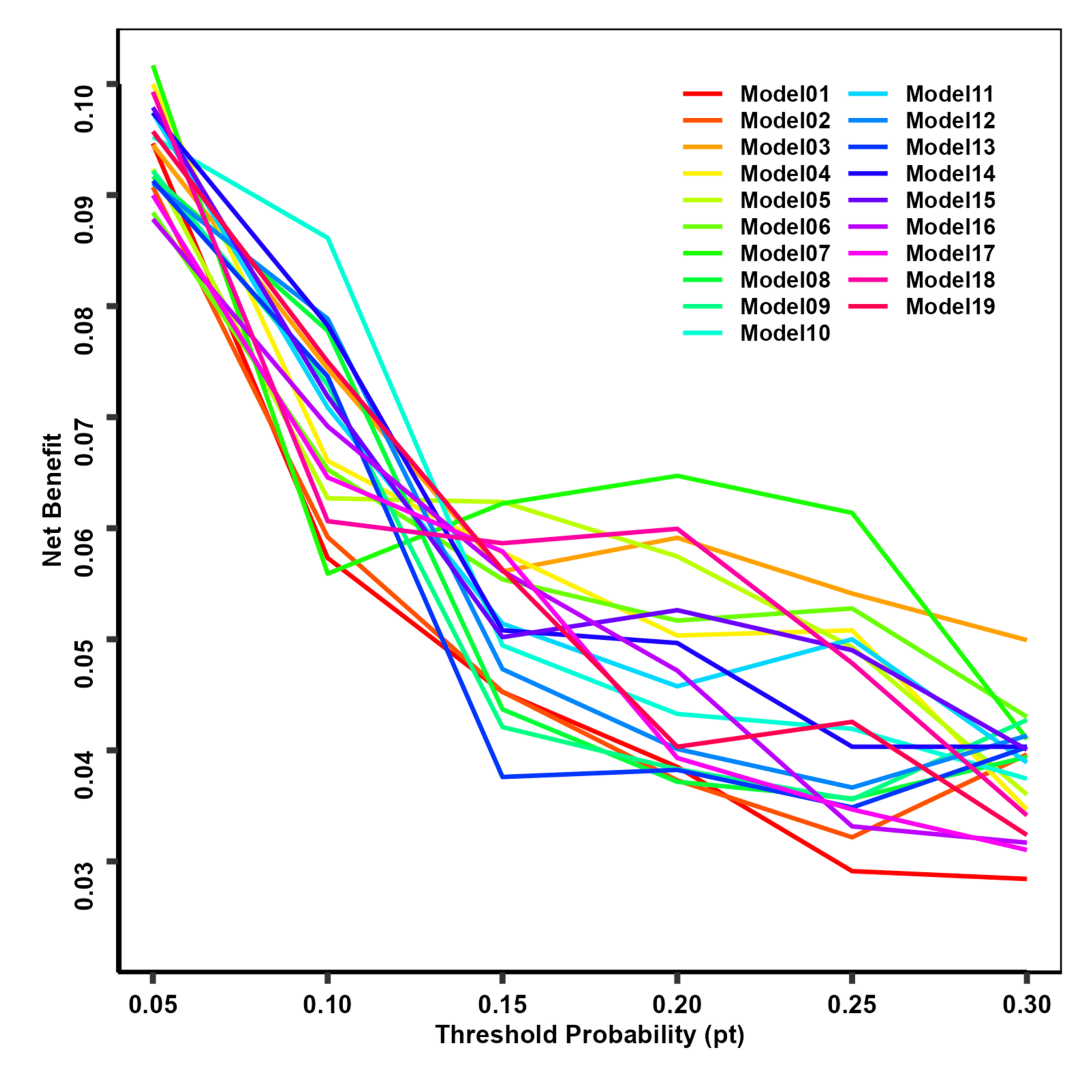
Decision curve analysis for all 19 AKI predictive models included in the internal validation. Decision curve analysis (DCA) was employed to evaluate the clinical utility (net benefit) of 19 predictive models for acute kidney injury (AKI) following transcatheter aortic valve implantation across a range of threshold probabilities. Model 1 includes the preoperative estimated glomerular filtration rate (eGFR) alone. Model 2 is a conventional clinical model consisting of eGFR, age, body mass index, and left ventricular ejection fraction. Model 3 is a full model incorporating all variables, including eGFR, urinary biomarkers (liver-type fatty acid–binding protein (L-FABP) and clusterin), serum albumin, ejection fraction (EF), C-reactive protein, and hemoglobin. Models 4-7 are simplified models combining eGFR with conventional clinical variables. Models 8-19 evaluate combinations of eGFR with urinary L-FABP and/or clusterin and a single clinical variable. Internal validation was performed using the bootstrap method. Model compositions are summarized in Table 4.

Although 17 candidate models were generated during the LASSO regression-based model development process, only two models (Models 10 and 11) were selected for primary reporting in this study based on clinical interpretability and methodological relevance. All the 19 models, including the reference and conventional models, and their full coefficient sets are provided in Table 4 and Figures 2-4.

**Table 4:** Results of logistic regression analyses for post-TAVI AKI *p<0.05. AKI vs. non-AKI group. AKI, acute kidney injury; TAVI, transcatheter aortic valve implantation; OR, odds ratio; CI, confidence interval; ACE, inhibitor angiotensin–converting enzyme inhibitor; ARB, angiotensin II receptor blocker; Cr, creatinine; eGFR, estimated glomerular filtration rate; WBC, white blood cell count; CRP, C-reactive protein; NT-pro BNP, N-terminal pro B-type natriuretic peptide; Log pre-L-FABP, Log preoperative liver-type fatty acid–binding protein; Log pre-clusterin, Log preoperative clusterin. eGFR was calculated using the Japanese coefficient–modified Chronic Kidney Disease Epidemiology Collaboration equation [13]: $\mathrm{eGFR}=194 \times (\mathrm{Creatinine})^{-1.094}\times (\mathrm{Age})^{-0.287}\times (0.739\ \text{if female})$

Variable	Unadjusted (Univariate)			Selected by LASSO
	OR	95%CI	P value	
Sex (M/F)	0.43	0.180-1.03	0.059	-
Age (years)	1.01	0.93-1.10	0.748	-
Body mass index (kg/m2)	1.09	0.975-1.23	0.123	-
Left ventricular ejection fraction (%)	0.959	0.926-0.994	0.021^*^	-
Mean gradient, mmHg	0.992	0.972-1.01	0.446	-
Hypertension, n (%)	∞	–	–	-
ACE inhibitor/ARB	0.915	0.384-2.18	0.842	-
Preoperative blood test results				
Hemoglobin Level (g/dL)	0.810	0.623-1.05	0.114	-
Serum albumin (g/dL)	0.169	0.068-0.420	< 0.001^*^	○
Serum sodium (mEq/L)	1.01	0.883-1.16	0.882	-
Serum potassium (mEq/L)	1.03	0.407-2.59	0.957	-
Serum chloride (mEq/L)	1.08	0.951-1.23	0.232	-
Serum Cr (mg/dL)	4.27	2.15-8.48	<0.001^*^	-
eGFR(8mL/min/1.73m²)	0.939	0.912-0.966	<0.001^*^	○
WBC count (×10³/µL)	1.14	0.939-1.39	0.183	-
CRP (mg/dL)	1.44	1.08-1.93	0.014^*^	○
NT-pro BNP (pg/mL)	1.00	1.00-1.00	0.006^*^	-
Preoperative urinary parameters				
Log pre L-FABP	2.20	1.51-3.20	<0.001^*^	○
Log pre Clusterin	1.90	1.43-2.53	<0.001^*^	○

Receiver operating characteristic curve analysis and diagnostic accuracy

**Figure 5:**
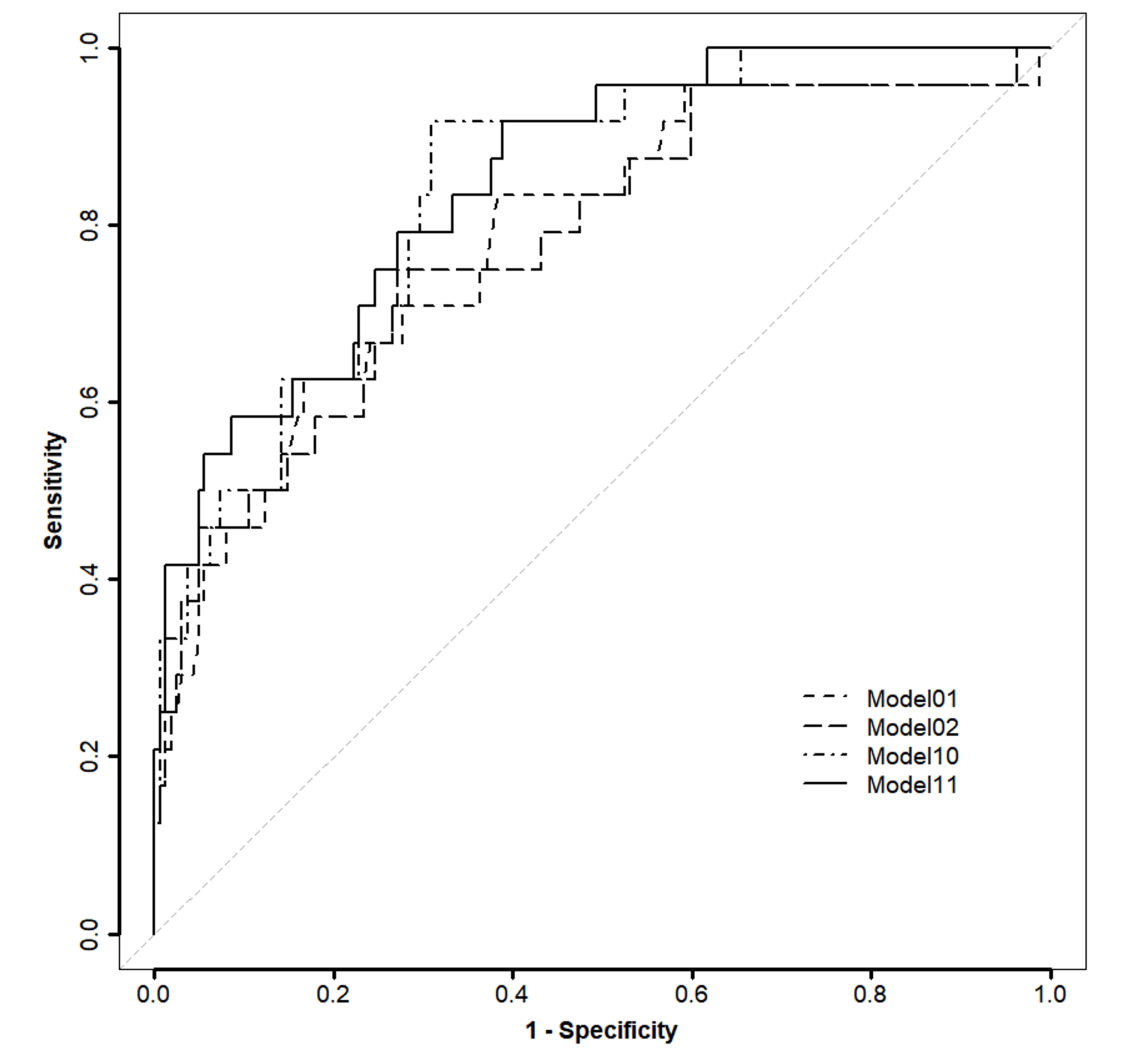
Receiver operating characteristic curves (ROC) for the internal validation of acute kidney injury (AKI) predictive models ROC curves for four AKI predictive models: Model 1 (estimated glomerular filtration rate (eGFR) alone, dashed line, control), Model 2 (eGFR+age+body mass index+ejection fraction, long dashed line, traditional model), Model 10 (eGFR+urinary liver-type fatty acid–binding protein (L-FABP)+clusterin+serum albumin, dash-dot line), and Model 11 (eGFR+L-FABP+clusterin+C-reactive protein, solid line). L-FABP and clusterin were log-transformed. Curves represent internal validation using 200 bootstrap resamples.

Table 5 summarizes the discriminative performance of individual predictors and combined models for post-TAVI AKI. Preoperative eGFR, log pre-L-FABP, log pre-clusterin, preoperative serum albumin, and pre-CRP showed AUCs of 0.78, 0.69, 0.76, 0.75, and 0.69, respectively. Among the combined models, those incorporating urinary biomarkers and clinical variables demonstrated superior predictive performance compared with the reference and conventional models (Models 1 and 2). Model 10, which included serum albumin in addition to urinary biomarkers and clinical variables, showed intermediate predictive performance (AUC=0.84). Model 11 showed the highest discriminative ability, with an AUC of 0.85 (Figure 5). Compared with Model 1, Model 11 also improved risk reclassification, with positive NRI (0.132) and IDI (0.143) values (Table 5).

**Table 5:** AUC, NRI, and IDI for the post-TAVI AKI prediction model Data are given as AUC (95% confidence interval). AUC, area under the curve; NRI, net reclassification improvement; IDI, integrated discrimination improvement; TAVI, transcatheter aortic valve implantation; ΔAUC (%) ΔAUC × 100/AUC_base; Pre-eGFR, preoperative estimated glomerular filtration rate; Log pre L-FABP, log preoperative liver-type fatty acid–binding protein; Log pre-Clusterin, log preoperative Clusterin; Pre-CRP, preoperative C-reactive protein; BMI, body mass index; EF, left ventricular ejection fraction; Pre-sAlb, preoperative serum albumin. eGFR was calculated using the Japanese coefficient–modified Chronic Kidney Disease Epidemiology Collaboration equation [13]: $\mathrm{eGFR}=194 \times (\mathrm{Creatinine})^{-1.094}\times (\mathrm{Age})^{-0.287}\times (0.739\ \text{if female})$

	AUC (unadjusted)	AUC (optimism- corrected)	C index	ΔAUC (%) (unadjusted)	ΔAUC (%) (optimism-corrected)	NRI	IDI
Pre-eGFR	0.78 (0.68-0.89)						
Log pre-L-FABP	0.69 (0.56-0.83)						
Log pre-Clusterin	0.76 (0.64-0.88)						
Pre-serum albumin	0.75 (0.64-0.85)						
Pre-CRP	0.69 (0.58-0.79)						
Model 1: Pre-eGFR	0.78 (0.68-0.89)	0.78 (0.78-0.79)	0.78	-	-	-	-
Model 2: Pre-eGFR+age+BMI+EF	0.78 (0.67-0.89)	0.75 (0.75-0.76)	0.75	-0.43	-2.95	0.064	0.016
Model 10: Pre-eGFR+Log pre-L-FABP+Log pre-Clusterin+Pre-sAlb	0.84 (0.76-0.92)	0.81 (0.80-0.82)	0.81	5.79	2.99	0.164	0.118
Model 11: Pre-eGFR+Log pre-L-FABP+Log pre-Clusterin+Pre-CRP	0.85 (0.77-0.93)	0.82 (0.81-0.83)	0.82	6.31	3.63	0.132	0.143

Calibration of the predictive model

**Figure 6:**
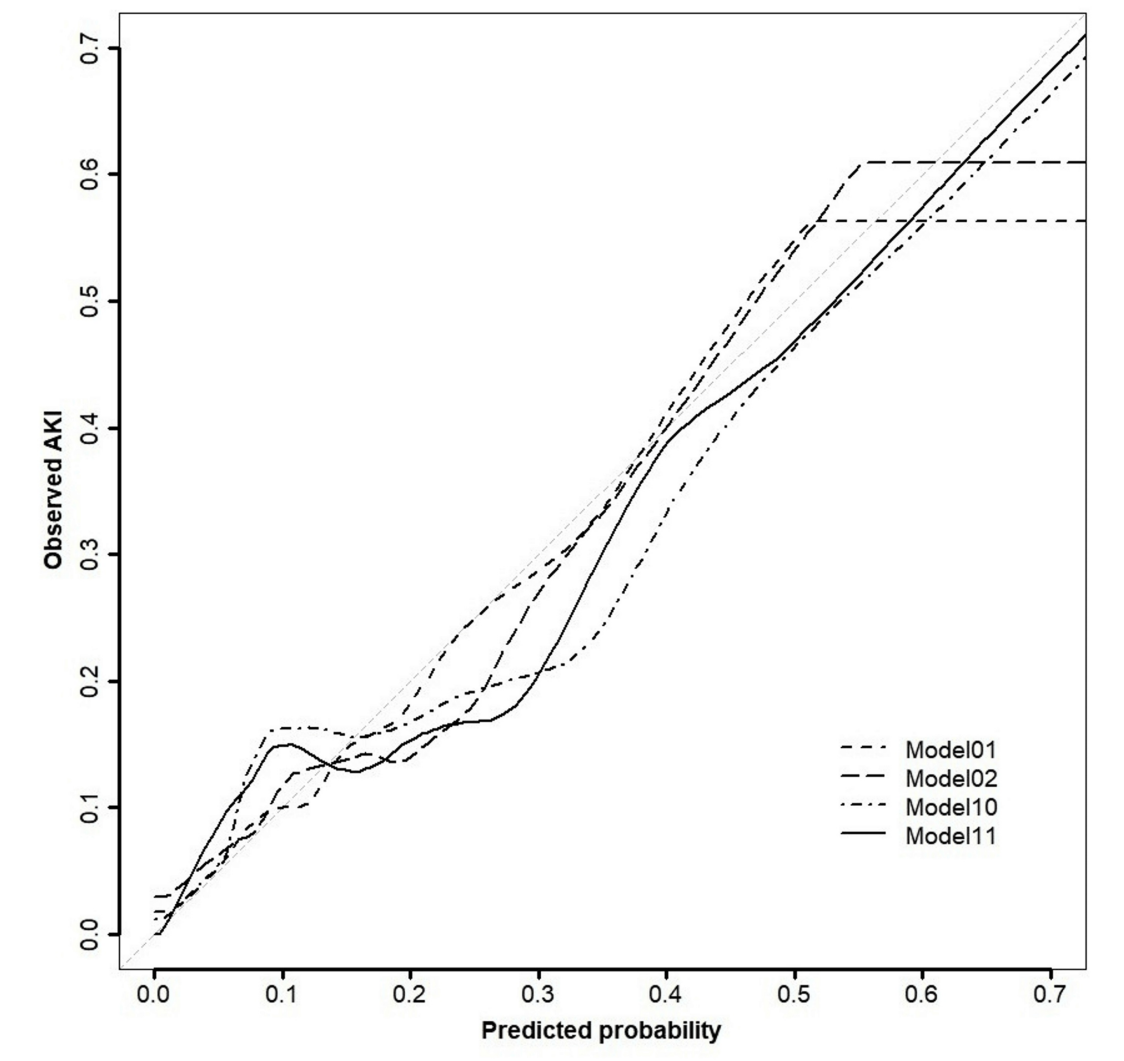
Calibration plots for the internal validation of acute kidney injury (AKI) predictive models Calibration plots for four AKI predictive models: Model 1 (estimated glomerular filtration rate [eGFR] alone, dashed line, control), Model 2 (eGFR+age+body mass index+ejection fraction, long dashed line, traditional model), Model 10 (eGFR+urinary liver-type fatty acid–binding protein (L-FABP)+clusterin+serum albumin, dash-dot line, main Model 1), and Model 11 (eGFR+L-FABP+clusterin+C-reactive protein, solid line, main Model 2). The thin diagonal solid line denotes perfect calibration (predicted=observed). L-FABP and clusterin were log-transformed. Plots represent internal validation using 200 bootstrap resamples.

Calibration of the best predictive model (Model 11) was evaluated using calibration plots (Figure 6). The predicted probabilities of post-TAVI AKI closely corresponded with the observed outcomes, exhibiting good agreement between the predicted and observed risks. The model’s C-index was 0.85 (Figure 5 and Table 5), indicating strong discriminative performance.

Clinical utility analysis

**Figure 7:**
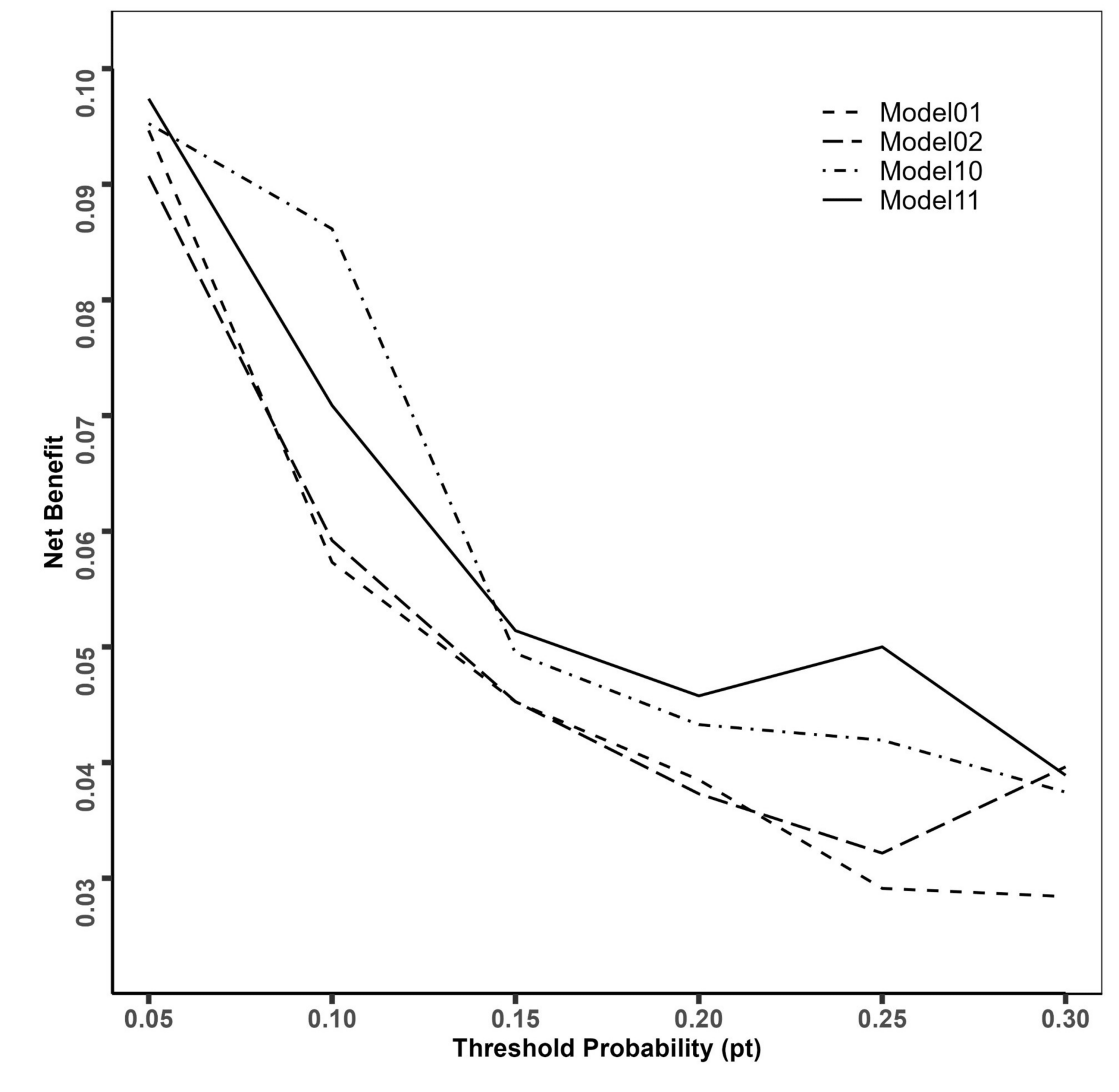
Decision curve analysis for the internal validation of acute kidney injury (AKI) predictive models Decision curves for four AKI predictive models: Model 1 (estimated glomerular filtration rate (eGFR) alone, dashed line, control), Model 2 (eGFR+age+BMI+ejection fraction, long dashed line, traditional model), Model 10 (eGFR+urinary L-FABP+clusterin+serum albumin, dash-dot line, main Model 1), and Model 11 (eGFR+L-FABP+clusterin+C-reactive protein, solid line, main Model 2). Curves for the “treat-all” and “treat-none” strategies are provided as references. L-FABP and clusterin were log-transformed. Curves represent internal validation using 200 bootstrap resamples.

In the DCA (Figure 7), Model 11 demonstrated the highest net benefit in the threshold probability of 0.05-0.30, with a notable advantage at lower thresholds (0.10-0.20). Models 1 and 2 showed a consistently low net benefit throughout this range.

## Discussion

The LASSO regression-based predictive model developed in this study demonstrated good predictive performance for post-TAVI AKI by combining preoperative urinary biomarkers (L-FABP and clusterin) with serum albumin [4,5], CRP [14,15], and eGFR [13]. These findings suggest that the development of post-TAVI AKI is determined not only by renal functional decline but also by multiple pathophysiological domains, including tubular injury, systemic inflammation, and nutritional and global physiological reserve [4,5,10,11,14,15].

Urinary L-FABP and clusterin are biomarkers reflecting tubular stress and injury. Both molecules have been reported as stress-response and injury-associated proteins in renal tubular cells [16,17]. L-FABP increases in response to enhanced fatty acid metabolism, hypoxia, and oxidative stress and is considered an early marker of tubular ischemia [16,17]. In contrast, clusterin is a stress-induced glycoprotein involved in cytoprotection and tissue repair processes and may reflect post-injury cellular remodeling [18,19]. These biomarkers represent distinct biological pathways, and their combined use enables a more comprehensive assessment of tubular injury. Their selection suggests the presence of subclinical tubular damage prior to changes in serum creatinine or eGFR, which may contribute to the development of post-TAVI AKI [9,12,16-19].

CRP was selected as a marker of systemic inflammation. Elevated CRP levels are associated with atherosclerosis, frailty, and endothelial dysfunction [14,15]. Chronic inflammatory status may increase the susceptibility of the kidney to perioperative insults such as ischemia-reperfusion injury and contrast exposure [11,20,21]. Therefore, CRP likely reflects an important inflammatory background contributing to AKI development in the perioperative setting.

Serum albumin was included as a marker of nutritional status and global physiological reserve (including hypoalbuminemia). Hypoalbuminemia has been associated not only with malnutrition and frailty but also with chronic inflammation and reduced physiological reserve [4,5]. In addition, albumin plays a role in maintaining oncotic pressure and endothelial stability; therefore, low albumin levels may increase susceptibility to renal hypoperfusion and ischemic injury.

A key feature of this study is that the predictive model was designed for preoperative risk stratification rather than post-injury diagnosis. In other words, the model aims to identify patients at high risk for AKI before the onset of clinically apparent renal dysfunction [6,8,22]. The final model, incorporating urinary L-FABP, clusterin, CRP, and eGFR, demonstrated good discriminative performance for post-TAVI AKI.

Each of the selected variables reflects a distinct pathophysiological domain: eGFR represents baseline renal functional reserve [13], CRP is an indicator of systemic inflammation [14,15], and L-FABP and clusterin provide an indication of tubular injury [16-19]. The integration of these complementary dimensions allows for a more comprehensive assessment of “latent renal vulnerability,” which cannot be captured by conventional clinical parameters alone [13,22]. Such preoperative risk stratification may provide supportive information for perioperative management strategies, including the application of KDIGO AKI care bundles, optimization of hemodynamic management, adjustment of contrast media use, nutritional support, and enhanced postoperative monitoring [6,8-11]. Furthermore, because this model integrates multiple domains associated with renal vulnerability, including renal functional reserve, tubular injury, systemic inflammatory status, and nutritional or physiological reserve, it may provide clinically useful information to help clinicians recognize patients who may warrant closer individualized perioperative management before the development of overt AKI. In this context, patients identified as high risk by this model may be considered for tailored preventive strategies, including careful evaluation of contrast exposure, optimization of hemodynamic management, enhanced renal function surveillance from the intraoperative period through the postoperative period, appropriate assessment of nutritional status, and avoidance of modifiable nephrotoxic factors. However, this study does not demonstrate that these interventions improve clinical outcomes.

Methodologically, the use of LASSO regression allowed for the selection of relevant predictors while minimizing overfitting in a dataset with a limited number of events [22]. Internal validation using bootstrap resampling suggested acceptable model stability and limited optimism bias [22]. Nevertheless, this study was a single-center retrospective analysis with a relatively small sample size; therefore, the findings should be interpreted as exploratory.

This study has several limitations. First, the model was developed utilizing data from a single institution, and its generalizability to other clinical settings or patient populations has yet to be established. Therefore, external validation using independent datasets is essential in future research. Second, the sample size was relatively small, with only 24 AKI events, which constrained model complexity and limited the feasibility of more sophisticated approaches. To alleviate overfitting under these conditions, we applied multivariable logistic regression and employed LASSO for predictor selection and shrinkage, alongside bootstrap resampling to obtain optimism-corrected estimates. However, meaningful subgroup analyses and more advanced machine learning approaches could not be performed. Third, although the model demonstrated good discriminative and calibration performance, its clinical interpretability may be limited, particularly with methods such as random forests. The contributions of individual predictors are not frequently intuitive, which may limit clinical applicability. Fourth, the retrospective nature of this study may have introduced variability in data quality and the timing of data collection. Some variables had missing values and were thus subjected to multiple imputation; however, this process may have influenced the model’s performance. In addition, patients with missing postoperative day 2 serum creatinine measurements were excluded from the analysis, which may have introduced selection bias. Fifth, although AKI was defined according to the KDIGO criteria, urine output data were incomplete, potentially influencing the accuracy of AKI staging. Altogether, these limitations highlight the exploratory nature of this pilot study. Given the single-center retrospective design and limited sample size, larger prospective multicenter studies with complete datasets and external validation are warranted to confirm the predictive performance, validity, and clinical utility of this model.

## Conclusions

The present study suggests that a LASSO regression-based predictive model incorporating preoperative urinary L-FABP and clusterin together with eGFR and CRP may improve preoperative risk stratification for post-TAVI acute kidney injury compared with models based on conventional clinical variables. This integrated approach may provide a more comprehensive assessment of latent renal vulnerability prior to TAVI by combining biomarkers of tubular stress with indicators of renal function and systemic inflammation.

## References

[1] Sudarsky D, Drutin Y, Kusniec F (2022). Acute kidney injury following transcatheter aortic valve implantation: association with contrast media dosage and contrast media based risk predication models. J Clin Med.

[2] Butala AD, Nanayakkara S, Navani RV (2024). Acute kidney injury following transcatheter aortic valve implantation - a contemporary perspective of incidence, predictors, and outcomes. Heart Lung Circ.

[3] Liao YB, Deng XX, Meng Y (2017). Predictors and outcome of acute kidney injury after transcatheter aortic valve implantation: a systematic review and meta-analysis. EuroIntervention.

[4] Gassa A, Borghardt JH, Maier J (2018). Effect of preoperative low serum albumin on postoperative complications and early mortality in patients undergoing transcatheter aortic valve replacement. J Thorac Dis.

[5] Wiedermann CJ, Wiedermann W, Joannidis M (2010). Hypoalbuminemia and acute kidney injury: a meta-analysis of observational clinical studies. Intensive Care Med.

[6] (2012). KDIGO Clinical Practice Guideline for Acute Kidney Injury. Kidney International Supplements.

[7] Petersen JK, Østergaard L, Carlson N (2024). Impact of acute kidney injury after transcatheter aortic valve replacement: a nationwide study. J Am Heart Assoc.

[8] Ostermann M, Zarbock A, Goldstein S (2020). Recommendations on acute kidney injury biomarkers from the acute disease quality initiative consensus conference: a consensus statement. JAMA Netw Open.

[9] Obata Y, Kamijo-Ikemori A, Shimmi S, Inoue S (2023). Clinical usefulness of urinary biomarkers for early prediction of acute kidney injury in patients undergoing transaortic valve implantation. Sci Rep.

[10] Basile DP, Anderson MD, Sutton TA (2012). Pathophysiology of acute kidney injury. Compr Physiol.

[11] Navaratnarajah A, Bhan A, Alcock E (2022). Systemic inflammation and oxidative stress contribute to acute kidney injury after transcatheter aortic valve implantation. Cardiol J.

[12] Obata Y, Kamijo-Ikemori A, Shimmi S, Seino Y, Inoue S (2025). Combination of urinary biomarkers for early detection of patients with acute kidney injury after transcatheter aortic valve implantation. J Cardiothorac Vasc Anesth.

[13] Matsuo S, Imai E, Horio M (2009). Revised equations for estimated GFR from serum creatinine in Japan. Am J Kidney Dis.

[14] Han SS, Kim DK, Kim S, Chin HJ, Chae DW, Na KY (2017). C-reactive protein predicts acute kidney injury and death after coronary artery bypass grafting. Ann Thorac Surg.

[15] Mohamed W, Asimakopoulos G (2021). Preoperative C-reactive protein as a predictor of postoperative acute kidney injury in patients undergoing coronary artery bypass grafting. Perfusion.

[16] Kamijo A, Sugaya T, Hikawa A (2004). Urinary excretion of fatty acid-binding protein reflects stress overload on the proximal tubules. Am J Pathol.

[17] Yamamoto T, Noiri E, Ono Y (2007). Renal L-type fatty acid-binding protein in acute ischemic injury. J Am Soc Nephrol.

[18] Jones SE, Jomary C (2002). Clusterin. Int J Biochem Cell Biol.

[19] Zhou W, Guan Q, Kwan CC, Chen H, Gleave ME, Nguan CY, Du C (2010). Loss of clusterin expression worsens renal ischemia-reperfusion injury. Am J Physiol Renal Physiol.

[20] Desanti De Oliveira B, Xu K, Shen TH (2019). Molecular nephrology: types of acute tubular injury. Nat Rev Nephrol.

[21] Seeliger E, Sendeski M, Rihal CS, Persson PB (2012). Contrast-induced kidney injury: mechanisms, risk factors, and prevention. Eur Heart J.

[22] Feng Y, Wang AY, Jun M (2023). Characterization of risk prediction models for acute kidney injury: a systematic review and meta-analysis. JAMA Netw Open.

